# Convergent transcriptomic and genomic evidence supporting a dysregulation of *CXCL16* and *CCL5* in Alzheimer’s disease

**DOI:** 10.1186/s13195-022-01159-5

**Published:** 2023-01-21

**Authors:** Xiao Li, Deng-Feng Zhang, Rui Bi, Li-Wen Tan, Xiaogang Chen, Min Xu, Yong-Gang Yao

**Affiliations:** 1 Key Laboratory of Animal Models and Human Disease Mechanisms of the Chinese Academy of Sciences & Yunnan Province, and KIZ/CUHK Joint Laboratory of Bioresources and Molecular Research in Common Diseases, Kunming Institute of Zoology, Chinese Academy of Sciences, Kunming, 650204 Yunnan China; 2 Kunming College of Life Science, University of Chinese Academy of Sciences, Kunming, 650204 China; 3 CAS Center for Excellence in Brain Science and Intelligence Technology, Chinese Academy of Sciences, Shanghai, 200031 China; 4 Mental Health Institute of the Second Xiangya Hospital, Central South University, Changsha, 410011 China

**Keywords:** Alzheimer’s disease, Chemokine, mRNA expression, Pathology, Targeted sequencing, Mendelian randomization

## Abstract

**Background:**

Neuroinflammatory factors, especially chemokines, have been widely reported to be involved in the pathogenesis of Alzheimer’s disease (AD). It is unclear how chemokines are altered in AD, and whether dysregulation of chemokines is the cause, or the consequence, of the disease.

**Methods:**

We initially screened the transcriptomic profiles of chemokines from publicly available datasets of brain tissues of AD patients and mouse models. Expression alteration of chemokines in the blood from AD patients was also measured to explore whether any chemokine might be used as a potential biomarker for AD. We further analyzed the association between the coding variants of chemokine genes and genetic susceptibility of AD by targeted sequencing of a Han Chinese case–control cohort. Mendelian randomization (MR) was performed to infer the causal association of chemokine dysregulation with AD development.

**Results:**

Three chemokine genes (*CCL5*, *CXCL1*, and *CXCL16*) were consistently upregulated in brain tissues from AD patients and the mouse models and were positively correlated with Aβ and tau pathology in AD mice. Peripheral blood mRNA expression of *CXCL16* was upregulated in mild cognitive impairment (MCI) and AD patients, indicating the potential of *CXCL16* as a biomarker for AD development. None of the coding variants within any chemokine gene conferred a genetic risk to AD. MR analysis confirmed a causal role of CCL5 dysregulation in AD mediated by trans-regulatory variants.

**Conclusions:**

In summary, we have provided transcriptomic and genomic evidence supporting an active role of dysregulated *CXCL16* and *CCL5* during AD development.

**Supplementary Information:**

The online version contains supplementary material available at 10.1186/s13195-022-01159-5.

## Background

Alzheimer’s disease (AD) is a progressive and incurable age-related neurodegenerative disease with cognitive decline caused by neuronal loss and brain atrophy [1, 2]. The neuropathological features of AD include the presence of abundant extracellular amyloid plaques laden with β-amyloid peptide (Aβ), intraneuronal neurofibrillary tangles formed by the hyperphosphorylated tau, neuritic dystrophy, loss of synapses and neuronal somata [1, 2]. Microglia activation and neuroinflammation are also hallmarks of AD [1, 2]. Genetic studies including genome-wide association studies (GWAS) [3–7], whole-exome sequencing (WES) [8, 9], and whole-genome sequencing (WGS) [10] studies have identified numerous genomic loci associated with AD, with immune genes being highlighted frequently.

The immune factors that are linked to neuroinflammation may accompany and contribute to neurodegenerative pathology [11]. Chemokines are small proteins with the ability to induce targeted chemotaxis of nearby reactive immune cells and have a well-established role in the immune system [12, 13]. Chemokines are a group of chemotactic cytokines and can be classified into two groups: the homeostatic chemokines and the inflammatory chemokines. The homeostatic chemokines are important for lymphoid organ development and immune cell trafficking. The inflammatory chemokines are actively involved in the mobilization of effector cells to the inflammatory sites [14]. Apart from their roles in the immune system, chemokines also take part in the physiological and pathological processes in the central nervous system (CNS). In the human brain, neurons and glial cells are able to express chemokines and also have chemokine receptors [15, 16]. Chemokines participate in the proliferation, differentiation, and migration of neural cells and are important for brain homeostasis [15]. The expression of chemokines and the functioning of their receptors may change in CNS diseases [14–16]. Chemokines have been reported to be actively involved in CNS development and neurological diseases [14]. Chemokine signaling affects a variety of cellular activities and functions, including the migration and survival of neuronal precursors [17], the migration and proliferation of oligodendrocyte progenitors [18], the maintenance of oligodendrocyte lineage, myelination, and white matter [19], the central synaptic transmission [20], glymphatic function and neuroinflammation [21], and aging-dependent neuronal regenerative decline [22].

There are several lines of evidence to show expression change, chemokine/chemokine receptor axis signaling, pathological correlation, and genetic regulation of chemokines, each take a part in the disease progression of AD. First, expression changes of chemokines have been implicated in the pathogenesis of AD. Some chemokines were reported to be significantly altered in brain tissues [23], cerebral microcirculation [24], cerebrospinal fluid (CSF) [25, 26], plasma [27] or peripheral blood cells [28, 29] of AD patients, and in a mouse model of AD [30]. Second, chemokine/chemokine receptor axes may affect AD pathologies in multiple ways. The impaired CCL2/CCR2 axis in blood-derived monocytes caused a deficit in cell migration in mild cognitive impairment (MCI) and AD patients [31]. Dysregulation of the CX3CL1/CX3CR1 axis has shown both neuroprotective and neurotoxic effects in different AD mouse models [32, 33]. Third, a growing body of evidence indicates the chemokine/chemokine receptor axes affect Aβ and tau pathologies [32, 34–36], glymphatic function, cognition, or neuroinflammation [21, 36] in AD mouse models. Despite the functional involvement, several genetic association studies focusing on a few chemokine genes showed no direct association between common genetic polymorphisms of chemokine genes and the genetic susceptibility to AD [13, 37–39]. However, one study has suggested that a haplotype of single nucleotide polymorphism (SNP) within a chemokine gene cluster may modify the age of onset of familial AD [40]. Since genetic variants play a role in the regulation and activation of chemokine/chemokine receptor signaling in AD [27], a systematic evaluation of the genetic variants and regulation of chemokines in AD is warranted.

**Fig. 1:**
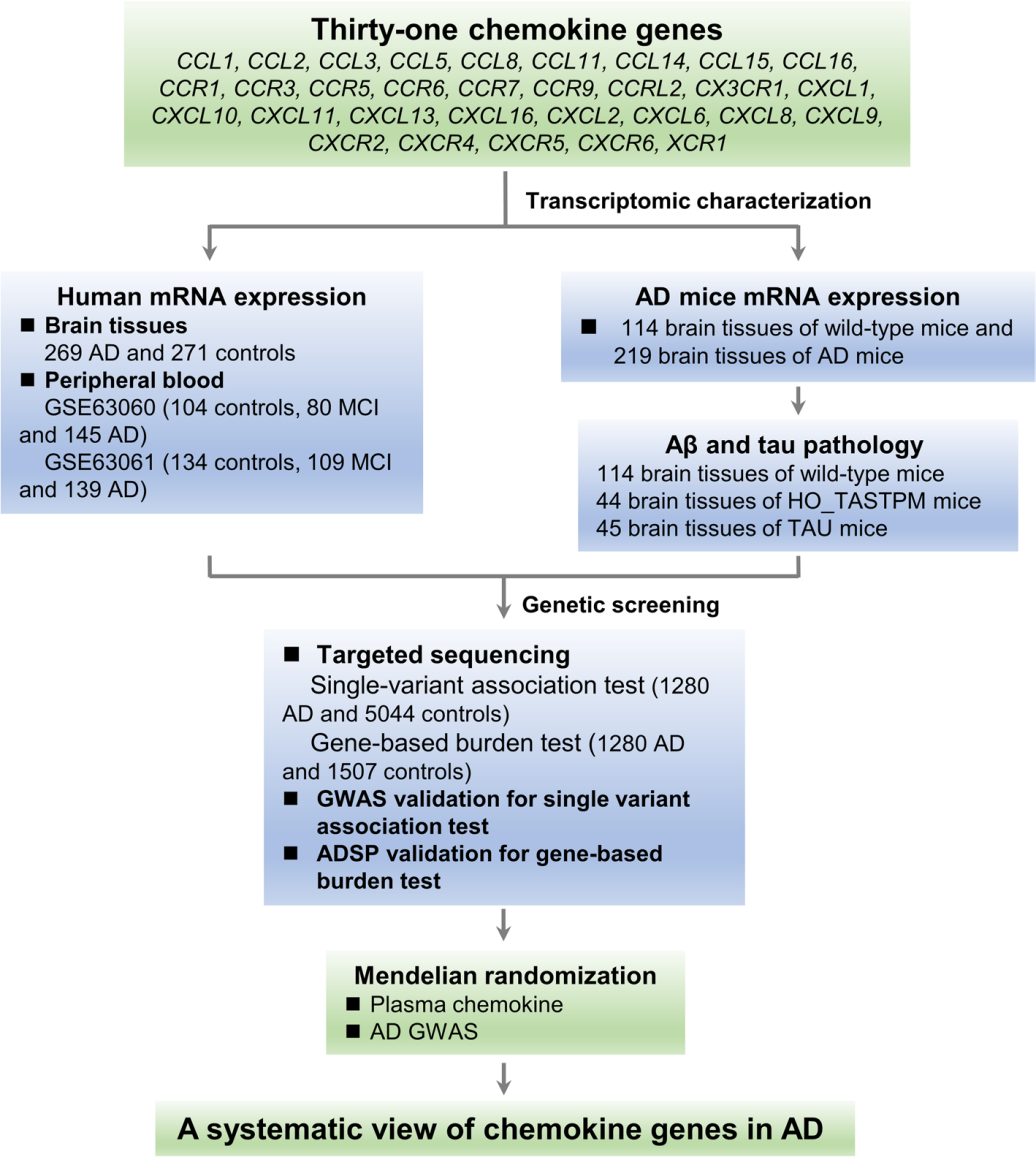
Study design for integrative analysis and for identifying AD-associated chemokines. The mRNA expression profiling of 31 chemokine genes was analyzed by using the compiled microarray data of four brain regions (entorhinal cortex, hippocampus, frontal cortex, and temporal cortex) of AD patients and controls [41], two microarray data (GSE63060 and GSE63061) from peripheral blood of patients with mild cognitive impairment (MCI) or AD and controls [42], and expression data of AD mouse models [43]. The gene-based burden test and single-variant association analysis were performed using Han Chinese cohorts in this study and reported datasets [6, 44]. Mendelian randomization (MR) was used to assess the causal effect of the most significantly AD-associated chemokine genes on AD

In this study, we evaluated the transcriptomic dysregulation of chemokines in publicly available datasets of AD patients and mouse models. A three-stage genetic study of 31 chemokine genes was conducted in a Han Chinese cohort of 1280 AD cases and 5044 cognitively normal control subjects, to evaluate the potential association of genetic variation of chemokines with AD. Mendelian randomization (MR) was further used to evaluate the causally associated chemokine gene(s) in AD (Fig. 1). We found an active involvement of *CXCL16* and *CCL5* dysregulation in the development of AD.

## Materials and methods

### Gene assignment

We assigned 31 typical chemokine genes, including nine C–C motif chemokine ligands (CCLs) and seven receptors (CCRs), nine C-X-C motif chemokine ligands (CXCLs) and four receptors (CXCRs), C-X3-C motif chemokine receptor (CX3CR1) and X-C motif chemokine receptor (XCR1), as defined by the KEGG “Chemokine signaling pathway” (https://www.gsea-msigdb.org/gsea/msigdb/cards/KEGG_CHEMOKINE_SIGNALING_PATHWAY.html) and the Immport database (https://www.immport.org/shared/genelists) [45].

### mRNA expression profiling of chemokine genes in AD patients

The mRNA expression levels of the chemokine genes were analyzed in four AD-relevant brain regions (the entorhinal cortex, hippocampus, temporal cortex and frontal cortex) [41]. The microarray expression data of the four brain regions from 269 AD patients and 271 controls were retrieved from Gene Expression Omnibus (GEO: http://www.ncbi.nlm.nih.gov/geo) and were integrated to generate a normalized expression profile, as described in our previous study (http://www.alzdata.org/) [41]. In brief, for each original microarray data retrieved from the GEO database, we conducted data normalization, log2 transformation, probe filtration, and probe mapping to entrez gene IDs. Expression datasets for the same brain region were then combined and re-normalized to remove batch effects. The normalized expression data was used to detect if there were any differentially expressed chemokine genes between AD patients and controls. More details regarding to data processing of these reported datasets can be retrieved from our previous study ([41] and references therein).

The expression alterations of chemokines in peripheral blood from individuals with and without AD were explored in two large independent age-matched dementia case–control data sets [42]. The first dataset (GSE63060) includes 329 individuals containing 104 healthy controls, 80 MCI, and 145 AD patients from AddneuroMed Cohort (batch 1, https://www.ncbi.nlm.nih.gov/geo/query/acc.cgi?acc=GSE63060). The second dataset (GSE63061) includes 388 individuals, among which 382 were explicitly defined (including 134 healthy controls, 109 MCI, and 139 AD patients) (batch 2, https://www.ncbi.nlm.nih.gov/geo/query/acc.cgi?acc=GSE63061) [42]. Patients with MCI and AD had different levels of cognitive impairments. Specifically, the MCI patients had problems with memory, but had normal daily activities. The healthy control subjects had no cognitive impairment. More description of these patients with AD, MCI, and healthy controls can be found in the original study [42]. The expression profiling of the original microarray expression matrix series was loaded using the R package *GEOquery*. The *limma* R package was used to process the data and differential expression analysis was conducted under the linear model. More information regarding the related datasets can be found in the original publication [42].

### mRNA expression profiling and pathological correlation in AD mouse models

So as to investigate the dynamic alteration of chemokines before and during the development of AD pathology, we retrieved the spatial–temporal expression data of chemokine genes in AD mouse models from Mouseac (www.mouseac.org) [43]. We retrieved pathological scores of Aβ and tau of the AD mouse models and performed the correlation analysis between the mRNA expression level of chemokine genes and scored AD pathology by using the nonparametric Pearson correlation test with the GraphPad Prism software (GraphPad Software, La Jolla, CA, USA), as described in our previous study [46]. The original study [43] measured the gene expression changes in the brain tissues (including 113 hippocampus samples, 113 cortex samples, and 111 cerebellum samples) from different AD murine models at the age of 2, 4, 8, and 18 months. Also, the levels of amyloid burden and phosphorylated tau pathology were investigated through immunostaining with antibodies for Aβ40 or phosphorylated tau, and the semi-quantitative scores were based on the pathology severity [43]. In total, the expression data and pathological features of 114 brain tissues from wild-type mice (WILD) and 219 brain tissues from five AD transgenic mice with human APP, PSEN1 or MAPT mutant (TAS10 [with APP ^K670N/M671L^ mutant], TPM [with PSEN1 ^M146V^ mutant], HO_TASTPM [with homozygous mutant of APP and PSEN1 mentioned above], HET_TASTPM [with heterozygous mutant of APP and PSEN1 mentioned above], and TAU [with MAPT ^P301L^ mutant]) were analyzed. More information about these mouse models can be found in the original research [43].

### Targeted sequencing of chemokine genes in Han Chinese

To investigate whether there is a potential genetic association between the chemokine gene variants and AD, we conducted targeted sequencing of 31 chemokine genes in Han Chinese with and without AD. The majority of Han Chinese samples have been described in our previous studies [46–49]. Briefly, two cohorts containing AD cases (*n* = 1280) and controls (*n* = 5044) were enrolled from Southern and Eastern China. All AD patients were confirmed to have no known pathogenic variants in *APP*, *PSEN1*, or *PSEN2*. The Southern cohort contains 635 sporadic AD patients (mean age 79.7 ± 8.2 years, 40.0% male) and 1507 controls (mean age 35.2 ± 15.5 years, 56.2% male) recruited from Sichuan, Hunan, and Yunnan Province of China. The Eastern cohort contains 645 AD patients (mean age 79.2 ± 9.1 years, 41.2% male) recruited from Shanghai and Zhejiang and 3537 controls from the general population with WGS data from the China Metabolic Analytics Project (ChinaMAP) [50]. The patients were diagnosed by at least two clinical psychiatrists following the Diagnostic and Statistical Manual of Mental Disorders (DSM-IV), as have been previously described [46–48]. Some of the control samples from the Yunnan Province of China have been reported in our previous study [51, 52]. The Southern and Eastern cohorts were used in stage 1 and stage 2 analyses, respectively. Written informed consents conforming to the tenets of the Declaration of Helsinki were obtained from all participants before the enrollment of this study. The experimental protocols were approved by the institutional review board of Kunming Institute of Zoology, Chinese Academy of Sciences.

Genomic DNA was extracted from peripheral blood by using the AxyPrep Blood Genomic DNA Miniprep Kit (Axygen Scientific). DNA probes were designed using the online NimbleDesign tool (now updated as https://www.hyperdesign.com/#/). Coding regions, exon–intron boundaries, the 5’- and 3’-untranslated regions (UTRs) of the 31 genes were captured with the NimbleGen SeqCap EZ Choice Enrichment Kit (Roche NimbleGen) according to the manufacturer’s protocols, as described in our recent studies [51, 52]. Briefly, all captured DNA libraries were sequenced with the NovaSeq 6000 (150 bp paired-end). Raw reads were trimmed to remove sequencing adapters and low-quality reads by using the Trimmomatic (v0.33) [53]. Clean reads were aligned according to the human reference genome GRCh37/hg19 with the Burrows-Wheeler Aligner [54]. Post alignment quality control and variant calling were performed using the Genome Analysis Toolkit (GATK v4.1) following the best practices pipeline (https://www.broadinstitute.org/gatk/guide/best-practices) [55]. Quality control was performed based on all of the sequencing data. Variants were excluded if the genotype rate were less than 90%, or deviated from Hardy–Weinberg equilibrium (HWE *p* < 1 × 10^−6^). Samples with an average genotype rate < 80% were also excluded.

Variants were annotated into different functional categories by using ANNOVAR [56]. A variant was defined as rare if it had a minor allele frequency (MAF) < 0.01, otherwise, it was defined as common. Rare variants were further classified into three categories: loss-of-function (LoF) variants, missense variants, and possibly pathogenic variants. Variants belonging to stop gain/loss, frameshift indels, initiation codon, and splice sites were defined as LoF variants. Rare missense variants with sensitivity score ≤ 0.95 were defined as possibly pathogenic by using the Mendelian Clinically Applicable Pathogenicity (M-CAP) [57].

### Cross-genetic validation in European-ancestry populations

Data from two previous studies [6, 44] were used for cross-validation of the association of the single variants and genes with AD identified in this study. The summary data of the newly published GWAS meta-analysis [6] contains a total of 111,326 clinically diagnosed/ “proxy” AD cases and 677,663 controls. We used this dataset to validate the potential association of common variants with AD in Han Chinese. The WES data from the discovery case–control association results of the Alzheimer’s Disease Sequencing Project (ADSP) [44] was used to validate the gene-based burden test discerned in this study. The cumulative minor allele counts from the 5740 AD cases and 5096 cognitively normal controls and the *p*-values adjusted for different covariates were extracted from the original study. More information regarding patient description and data quality control can be found in the original reports [6, 44].

### MR analyses

In order to explore whether there was a causal effect of the observed association between chemokine and AD, we conducted MR analyses by using the R package “TwoSampleMR” [58]. The causal effect of chemokine on AD was measured using chemokine as the exposure and AD as the outcome. Genetic variants (SNPs) associated with chemokine expression (*p*-value < 1 × 10^−5^) were set as the instrumental variables and were investigated in GWAS of outcomes. The causal effect of chemokine-related instruments (significant SNPs) on AD outcomes was assessed in large-scale GWAS for AD. In brief, we extracted plasma protein data for chemokines from 3301 healthy blood donors [59] and 3394 individuals with multiple cardiovascular diseases [60]. GWAS data for AD were extracted from the stage 1 summary results of 21,982 diagnosed AD cases and 41,944 controls in a large GWAS meta-analysis study [3]. Data were retrieved from IEU GWAS database (https://gwas.mrcieu.ac.uk/) [58] using the gwasglue R package (https://mrcieu.github.io/gwasglue/).

In consideration of the fact that reverse causation is a common confounding factor in observational studies, we also checked the reverse causal effect setting AD as the exposure and chemokine as the outcome in the MR analyses. SNPs associated with AD (*p*-value < 1 × 10^−5^) were used as the instrumental variables. The effect of the instruments was estimated in the corresponding GWAS for each outcome with inverse-variance weighted (IVW) linear regression [58]. For the significance of the MR effect, a MR *p*-value < 0.05 was defined as significant. All MR analyses were conducted in R version 3.6.3 with packages.

### Statistical analyses

We used Quanto software to evaluate the statistical power of our samples under the gene-only hypothesis and log additive model [61]. Fisher’s exact test was applied to test if the allele frequency of a variant was significantly different between AD patients and controls. Meta-analyses combined the Southern and Eastern cohorts were performed using the metafor R package under the fixed effects model [62]. Nominal significance was defined as *p* < 0.05. The Bonferroni correction for multiple testing was performed based on the corresponding numbers of tested variants or genes. Rare variants within each gene were arranged into a gene-based burden test. Single rare and common variants were subjected to association analysis. The Bonferroni corrected significance requires a *p* < 0.0016 (0.05/31 genes) for gene-based analysis, *p* < 5.96 × 10^−5^ (0.05/839 rare variants identified in this study) for rare variant association analysis, and *p* < 7.04 × 10^−4^ (0.05/71 common variants identified in this study) for common variant association analysis. The burden of rare variants in each targeted gene was tested by using the optimized sequence kernel association test (SKAT-O) in the R package SKAT [63]. Rare variants classified into LoF, possibly pathogenic, or missense in each gene, were assessed. As we had no detailed genotype data of each individual from the ChinaMAP [50], we followed the same strategy as in our recent study [52] and performed the burden tests using only the 1507 healthy individuals from the Southern cohort as the control sample in the burden test.

Two-tailed Student’s *t*-test was used to investigate the mRNA expression difference between AD patients and controls with the GraphPad Prism software. Correlations between mRNA levels of the chemokine genes and AD pathology scores from Mouseac [43] were measured by using the nonparametric Pearson correlation test with the GraphPad Prism software.

## Results

### Upregulation of mRNA levels of chemokine genes in AD brain tissues

**Fig. 2:**
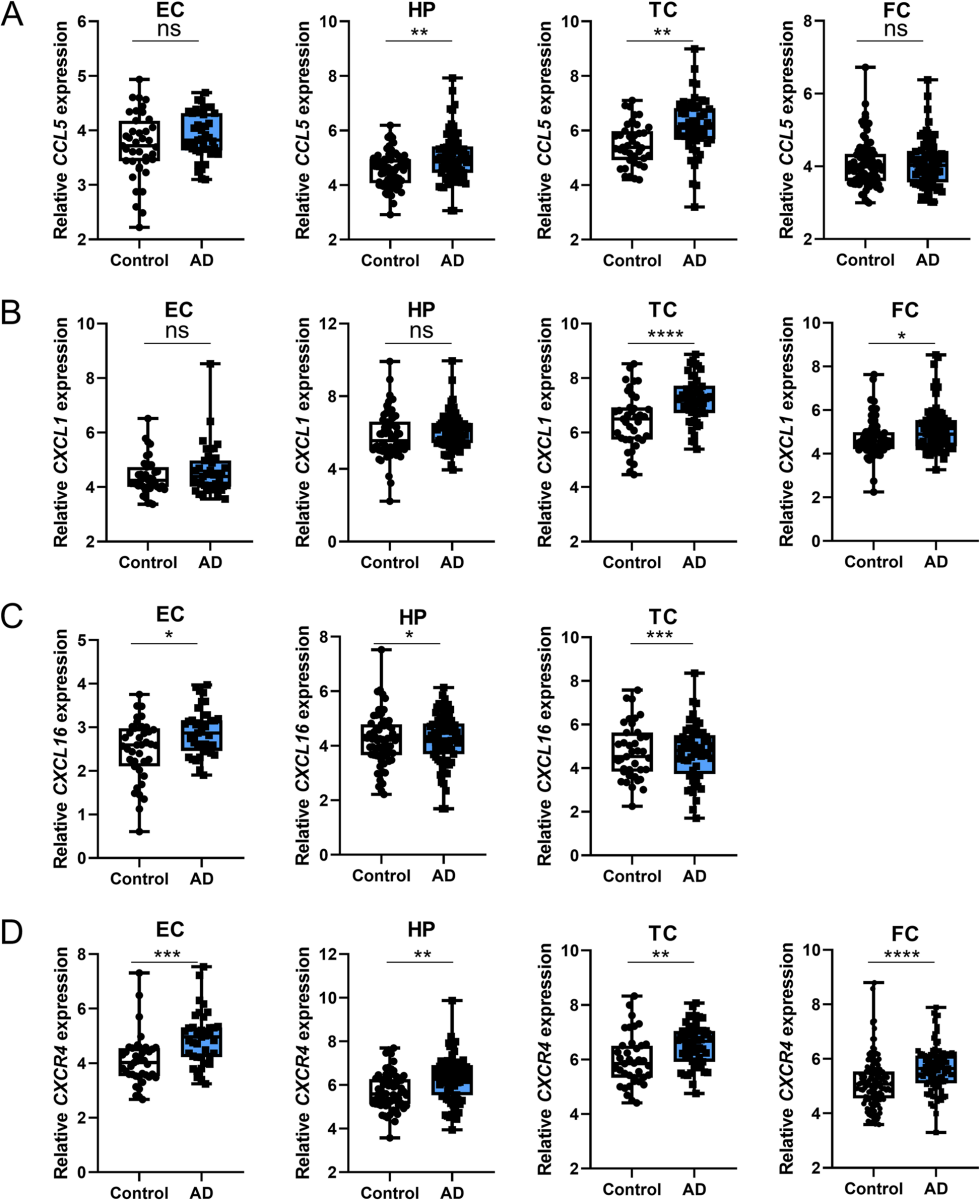
Upregulated mRNA expression levels of chemokine genes in brain tissues of AD patients. **A**–**D** The mRNA expression data were retrieved from the AlzData (www.alzdata.org) [41]. Data of *CXCL16* was not available in the frontal cortex tissues. Data from min to max were presented by dots. The lower and upper hinges of the boxes represent the first and third quantiles, the whiskers extend from min to max, and the line represents the median. EC, entorhinal cortex; HP, hippocampus; TC, temporal cortex; FC, frontal cortex; Ns, not significant. *, *P* < 0.05; **, *P* < 0.01; ***, *P* < 0.001; ****, *P* < 0.0001; two-tailed Student’s *t*-test

Since expression changes of chemokines are implicated in the pathogenesis of AD [32], we compared the mRNA expression levels of chemokine genes in four brain tissues (entorhinal cortex, hippocampus, temporal cortex, and frontal cortex) between AD patients and controls, using the normalized microarray data compiled in our previous study [41]. Among these 31 chemokine genes, no expression data of *CCL3*, *CCL14*, and *CCL15* were available for the analysis. The mRNA expression levels of 15 chemokine genes were nominally upregulated in one or more of the four AD-relevant tissues. Four genes, *CCL5*, *CXCL1*, *CXCL16*, and *CXCR4*, survived multiple-testing and showed significant differential expression (Table 1 and Fig. 2), suggesting a robust dysregulation of these genes in AD. No significant alteration of mRNA expression was observed for the remaining 13 genes.

**Table 1:** mRNA expression levels of chemokine genes in brain tissues from 269 AD patients and 271 controls

**Gene**	**Entorhinal cortex**		**Hippocampus**		**Temporal cortex**		**Frontal cortex**	
	**logFC**	***P***	**logFC**	***P***	**logFC**	***P***	**logFC**	***P***
C–C motif chemokine ligand								
*CCL1*	− 0.13	0.413	0.09	0.498	− 0.28	0.229	0.02	0.82
*CCL2*	0.68	0.021*	0.63	0.007*	0.52	0.094	0.19	0.351
*CCL5*	0.17	0.17	0.46	0.001*	0.64	0.001*	0.02	0.841
*CCL8*	0.43	0.017*	0.32	0.081	0.12	0.619	− 0.19	0.066
*CCL11*	0.23	0.128	− 0.16	0.213	0.19	0.409	0.01	0.901
*CCL16*	0.18	0.194	0.29	0.056	− 0.07	0.785	− 0.13	0.211
C–C motif chemokine receptor								
*CCR1*	0.28	0.127	0.32	0.006*	0.4	0.008*	0.26	0.008*
*CCR3*	0.11	0.398	0.22	0.143	0.01	0.973	− 0.06	0.61
*CCR5*	NA	NA	0.38	0.006*	NA	NA	− 0.02	0.864
*CCR6*	0.12	0.411	− 0.02	0.892	− 0.18	0.464	− 0.08	0.46
*CCR7*	0.32	0.034*	0.15	0.279	− 0.09	0.748	0.07	0.398
*CCR9*	0.27	0.036*	− 0.08	0.61	− 0.04	0.838	− 0.04	0.692
*CCRL2*	0.18	0.206	NA	NA	0.28	0.207	− 0.01	0.923
C-X-C motif chemokine ligand								
*CXCL1*	0.21	0.263	0.26	0.18	0.79	**8.89 × 10**^−5^*	0.39	0.012*
*CXCL2*	0.23	0.192	0.2	0.257	0.17	0.405	0.09	0.483
*CXCL6*	0.41	0.005*	0.04	0.805	− 0.07	0.807	− 0.06	0.635
*CXCL8*	0.28	0.274	0.53	0.004*	− 0.06	0.729	0.03	0.816
*CXCL9*	0.31	0.016*	− 0.16	0.281	0.43	0.07	0.2	0.133
*CXCL10*	0.45	0.097	0.35	0.168	0.71	0.007*	0.41	0.026*
*CXCL11*	0.24	0.165	0.12	0.406	− 0.1	0.691	0.17	0.275
*CXCL13*	0.16	0.289	− 0.06	0.655	− 0.09	0.754	− 0.03	0.752
*CXCL16*	0.38	0.011*	0.29	0.012*	0.64	**2.16 × 10**^−4^*	NA	NA
C-X-C motif chemokine receptor								
*CXCR2*	NA	NA	0.26	0.088	− 0.05	0.849	− 0.03	0.847
*CXCR4*	0.8	**3.3 × 10**^−4^*	0.56	0.001*	0.55	0.003*	0.5	**3.79 × 10**^−5^*
*CXCR5*	0.03	0.776	0.11	0.32	0.08	0.712	− 0.12	0.22
*CXCR6*	0.14	0.245	0.16	0.091	0.16	0.278	0.2	0.011*
C-X3-C motif chemokine receptor								
*CX3CR1*	0.06	0.759	− 0.09	0.589	− 0.12	0.535	0.08	0.569
X-C motif chemokine receptor								
*XCR1*	0.1	0.392	0	0.977	0.09	0.637	0.01	0.884

The mRNA expression data were retrieved from the AlzData database (www.alzdata.org) [41], with no data available for *CCL3*, *CCL14*, and *CCL15*

A *p* value < 0.05 was marked with “*,” and a *p* value < 0.0018 after Bonferroni correction for the number of tested genes (0.05/28) was marked in bold

LogFC, log2 fold change of mRNA expressional mean value in AD patients relative to that in controls

### Correlation of chemokine mRNA expression levels with Aβ and tau pathology in AD mouse models

We reanalyzed the expression data of the AD mouse models and compared the gene expression levels of chemokines in brain tissues including 114 from WILD mice, 44 from HO_TASTPM mice, and 45 from TAU mice at different ages [43]. The mRNA expression levels of 10 chemokine genes (*Ccl3*, *Ccl5*, *Ccl8*, *Ccrl2*, *Cx3cr1*, *Cxcl1*, *Cxcl9*, *Cxcl10*, *Cxcl13*, and *Cxcl16*) were upregulated along with age and reached a significant level of differential expression at the late stage of AD. Among these 10 chemokines, six genes (*Ccl3*, *Ccl5*, *Cxcl1*, *Cxcl10*, *Cxcl13*, and *Cxcl16*) were upregulated in both HO_TASTPM and TAU mice (Additional file 1: Fig. S1). We observed a strong positive correlation between the mRNA expression of the above 10 genes and AD pathology. Except for the *Ccrl2* gene, whose mRNA expression was positively correlated only with Aβ pathology, the other nine genes showed a positive correlation of mRNA expression with both Aβ and tau pathology (Additional file 1: Fig. S2). These observations support an active involvement of chemokines in AD pathology in mouse models.

**Fig. 3:**
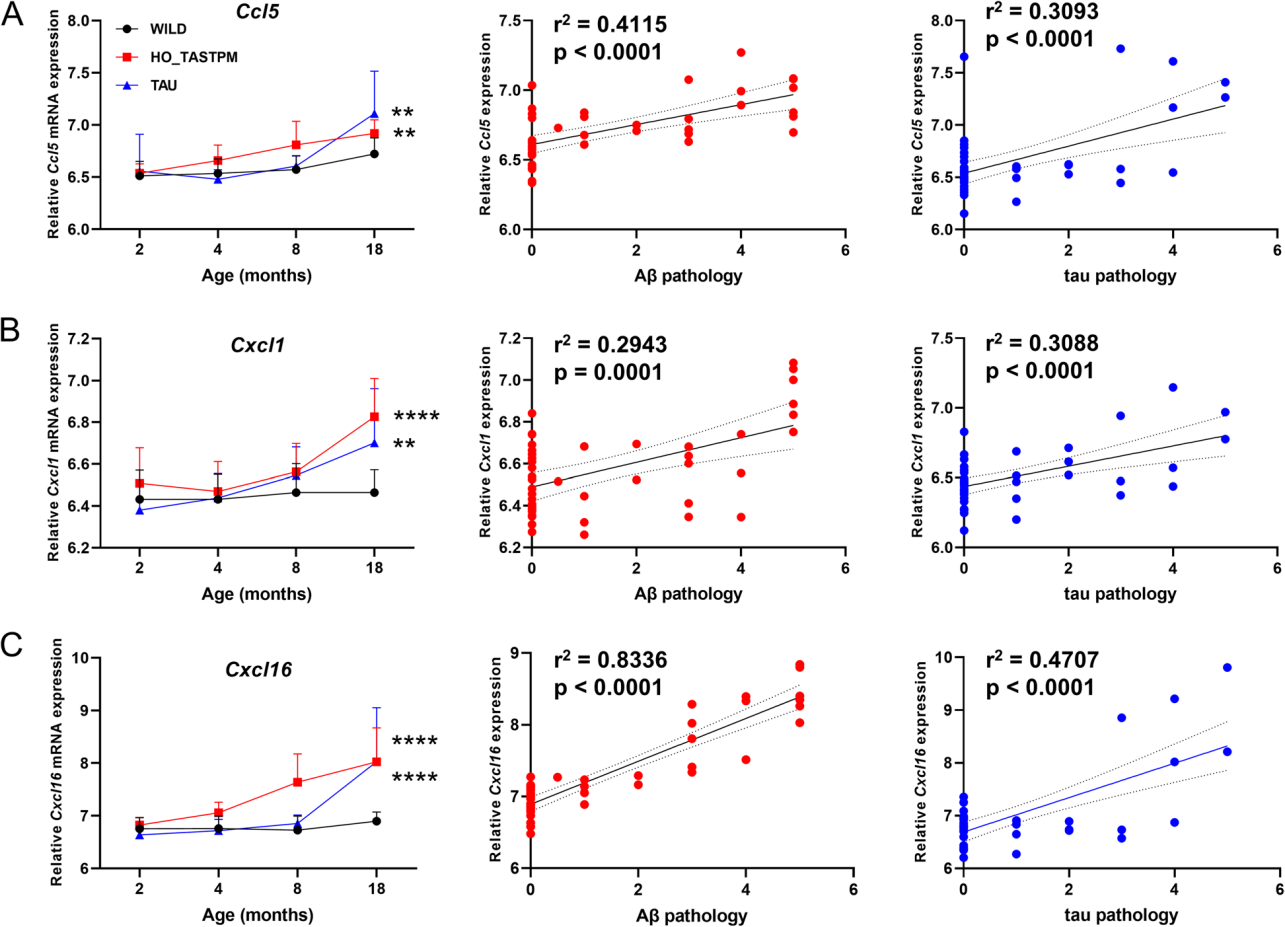
Correlation of upregulated mRNA levels of *Ccl5* (**A**), *Cxcl1* (**B**), and *Cxcl16* (**C**) with Aβ and tau pathology in AD mouse models. Original data were retrieved from Mouseac (www.mouseac.org) [43]. The age-related mRNA expression level was measured in 114 brain tissues from wild-type mice (WILD), 44 brain tissues from homozygous APP/PSEN1 double mutant mice (HO_TASTPM), and 45 brain tissues from mutant human MAPT mice (TAU) at different ages. The scores of Aβ pathology and tau pathology were based on 44 brain tissues of HO_TASTPM mice and 45 brain tissues of TAU mice, respectively. Error bars represent the population standard deviation. **, *P* < 0.01; ****, *P* < 0.0001; two-tailed Student’s *t*-test for comparison of mRNA expression between AD transgenic mice and WILD mice at month 18. The correlation between mRNA expression levels and pathology was measured using the Pearson correlation analysis. The solid and dashed lines represent the slope and the 95% confidence intervals in linear regression

We overlapped the genes with significant expression alteration in brain tissues from AD patients and the mouse models, as we speculated that these genes validated in two systems may be more reliable for being causal for AD development. Three genes, including *CCL5*, *CXCL1*, and *CXCL16* showed a consistent alteration pattern in AD patients and in the mouse models (Figs. 2 and 3).

### Upregulation of *CXCL16* expression in peripheral blood of patients with MCI and AD

**Fig. 4:**
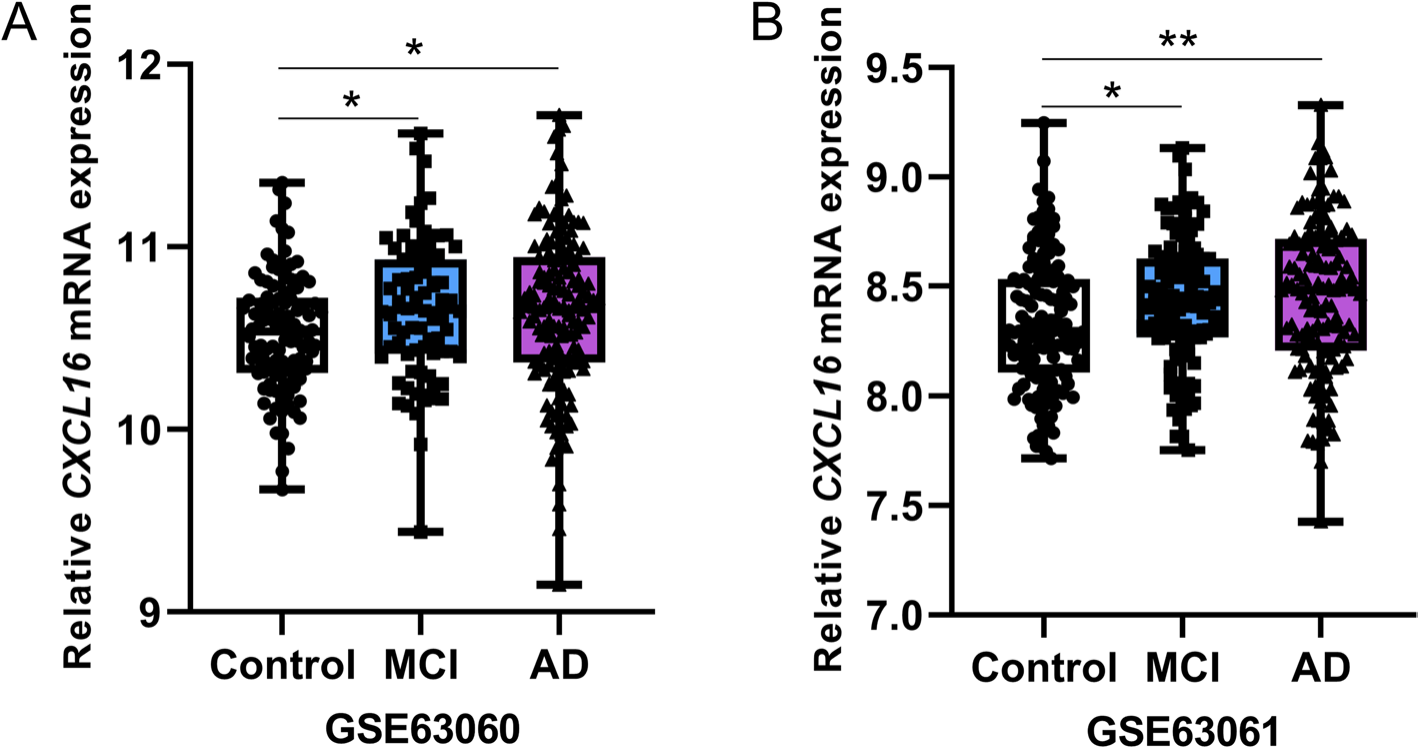
Upregulated mRNA expression of *CXCL16* in peripheral blood of patients with MCI and AD. Datasets of (**A**) GSE63060 (104 controls, 80 MCI, and 145 AD patients) and (**B**) GSE63061 (134 controls, 109 MCI, and 139 AD patients) [42] were used for determining *CXCL16* mRNA expression levels. Data from min to max were presented by dots. *, *P* < 0.05; **, *P* < 0.01; two-tailed Student’s *t*-test

To investigate if any of these dysregulated chemokine genes could be used as a potential biomarker for AD development, we analyzed the mRNA expression alterations of these genes in the peripheral blood from MCI patients, AD patients, and healthy controls. Among the three genes (*CCL5*, *CXCL1*, and *CXCL16*) consistently upregulated in brain tissues of AD patients and the mouse models, only *CXCL16* showed an upregulated expression in peripheral blood of MCI and AD patients as compared to controls (Table 2 and Fig. 4). This result suggests the potential of *CXCL16* to be a biomarker for AD development.

**Table 2:** mRNA expression levels of chemokine genes in peripheral blood of patients with MCI and AD and healthy controls

**Gene**	**ID (GSE63060)**	**80 MCI/104 controls**		**145 AD/104 controls**		**ID (GSE63061)**	**109 MCI/134 controls**		**139 AD/134 controls**	
		**logFC**	***P***	**logFC**	***P***		**logFC**	***P***	**logFC**	***P***
C–C motif chemokine ligand										
*CCL2*	ILMN_1720048	− 0.013	0.210	0.003	0.793	ILMN_1720048	0.005	0.416	− 0.002	0.670
*CCL3*	ILMN_1671509	0.043	0.012*	0.050	6.65 × 10^−4^*	ILMN_1671509	0.006	0.647	0.015	0.216
*CCL5*	ILMN_1773352	0.086	0.241	6.75 × 10^−4^	0.991	ILMN_1773352	0.011	0.853	0.032	0.575
*CCL8*	ILMN_1772964	0.038	0.036*	0.016	0.291	ILMN_1772964	0.029	0.482	− 0.017	0.650
*CCL11*	ILMN_1725519	0.002	0.877	0.002	0.806	-	-	-	-	-
*CCL14*	-	-	-	-	-	ILMN_3192001	− 6.72 × 10^−4^	0.895	0.003	0.6
*CCL15*	ILMN_1670658	− 0.002	0.809	− 0.009	0.250	-	-	-	-	-
*CCL16*	ILMN_2045324	0.004	0.677	− 7.10 × 10^−4^	0.935	-	-	-	-	-
C–C motif chemokine receptor										
*CCR1*	ILMN_1678833	− 0.166	0.001*	− 0.037	0.420	ILMN_1678833	− 0.039	0.319	− 0.033	0.370
*CCR3*	ILMN_1763322	0.027	0.741	− 0.085	0.219	ILMN_1763322	0.104	0.130	− 0.032	0.601
*CCR5*	ILMN_2145033	− 0.012	0.379	− 0.006	0.609	ILMN_2145033	− 0.001	0.795	0.004	0.464
*CCR6*	ILMN_2387696	0.054	0.084	0.034	0.189	ILMN_2387696	0.017	0.496	0.027	0.246
*CCR7*	ILMN_1715131	0.030	0.715	0.026	0.733	ILMN_1715131	− 0.11	0.094	− 0.092	0.166
*CCR9*	ILMN_2337386	− 0.008	0.561	0.005	0.737	ILMN_2337386	− 0.006	0.176	− 0.005	0.222
*CCRL2*	ILMN_1675346	− 0.015	0.387	− 0.012	0.435	ILMN_1675346	0.001	0.804	− 0.007	0.107
C-X-C motif chemokine ligand										
*CXCL1*	ILMN_1787897	− 0.007	0.599	0.002	0.841	ILMN_1787897	− 0.021	0.241	0.013	0.448
*CXCL6*	ILMN_2161577	− 0.006	0.447	0.011	0.116	ILMN_2161577	− 0.003	0.411	− 7.16 × 10^−5^	0.986
*CXCL8*	ILMN_1666733	− 0.047	0.339	0.075	0.092	ILMN_1666733	− 0.025	0.310	− 0.004	0.875
*CXCL9*	ILMN_1745356	0.006	0.611	0.011	0.291	-	-	-	-	-
*CXCL10*	ILMN_1791759	− 0.119	8.36 × 10^−5^*	− 0.046	0.138	ILMN_1791759	− 0.034	0.043*	− 0.036	0.025*
*CXCL11*	ILMN_2067890	0.010	0.270	0.002	0.809	ILMN_2067890	5.29 × 10^−3^	0.229	0.006	0.168
*CXCL13*	ILMN_1718552	− 0.014	0.136	− 0.004	0.638	ILMN_1718552	0.002	0.637	− 0.002	0.597
*CXCL16*	ILMN_1728478	0.124	0.017*	0.111	0.029*	ILMN_1728478	0.097	0.012*	0.118	0.003*
C-X-C motif chemokine receptor										
*CXCR2*	ILMN_1680397	0.264	2.30 × 10^−5^*	0.155	0.037*	ILMN_1680397	0.158	0.010*	0.122	0.057
*CXCR4*	ILMN_2320888	0.083	0.066	0.165	3.79 × 10^−5^*	ILMN_2320888	0.007	0.818	0.057	0.045
*CXCR5*	ILMN_2337928	0.069	0.321	− 0.033	0.556	ILMN_2337928	− 0.046	0.334	− 0.11	0.01
*CXCR6*	ILMN_1674640	− 0.003	0.902	0.004	0.864	ILMN_1674640	0.017	0.394	0.032	0.076
C-X3-C motif chemokine receptor										
*CX3CR1*	ILMN_1745788	0.067	0.098	0.013	0.716	ILMN_1745788	0.046	0.126	0.044	0.116
X-C motif chemokine receptor										
*XCR1*	ILMN_1764034	− 0.006	0.637	− 0.017	0.155	ILMN_1764034	0.008	0.416	0.008	0.323

The original data GSE63060 and GSE63061 [42] were extracted from GEO (https://www.ncbi.nlm.nih.gov/geo), with no data available for *CCL1* and *CXCL2*

Six individuals without explicit disease definition in GSE63061 were excluded from the analysis

A *p* value < 0.05 was marked with a “*”

-, missing data

*ID* Unique identifier for the probe

*LogFC* Log2 fold change of mRNA expressional mean value in MCI or AD patients relative to that in controls

### No association of chemokine genetic variants with AD in Han Chinese

Among these Han Chinese subjected to targeted sequencing, the mean sequencing depth of each gene was higher than 90 × in targeted sequencing (Additional file 1: Table S1), and the sequencing depth for each variant was higher than 25 × (Additional files 2 and 3). A total of 910 genetic variants (including 839 rare and 71 common variants) in 31 chemokine genes passed the quality control and were analyzed subsequently. The SKAT-O analysis [63] was conducted using rare variants in each gene for each cohort. In the Southern cohort, possibly pathogenic variants in *CCR7* (*p* = 0.004) and missense variants in *CCR9* (*p* = 0.009) were enriched in AD patients. In the Eastern cohort, *CCL3*, *CCR5*, and *CCR9* showed an association with AD at the gene-burden level. When combined both cohorts together to achieve a better statistic power, we found that five genes (including *CCL3*, *CCR7*, *CCR9*, *CCRL2*, and *XCR1*) were associated with AD at the gene-burden level and had an enrichment of rare variants (Additional file 1: Table S2). However, none of these significances survived the multiple testing correction, partially due to the limited sample size.

To confirm the gene-based association between chemokine genes and AD identified in Han Chinese, we used the WES data in the discovery stage of ADSP (5740 AD cases and 5096 cognitively normal controls) [44] as a validation cohort. None of these chemokine genes associated with AD in our Han Chinese population could be validated in the ADSP dataset [44], although we found that four genes (*CCL2*, *CCR6*, *CXCL6*, *CX3CR1*) were associated with AD at the gene-burden level under correction for different covariates, including the principal components, sequencing center, sex, age, and *APOE* ε4 and ε2 dosages, with the SKAT-O analysis [63] by using the ADSP dataset [44] (Additional file 1: Table S3).

Next, we tested the single-variant association of 839 rare and 71 common variants with AD risk in each Han Chinese cohort and the combined sample. We observed nominally significant association with AD of eight rare variants in six of the 31 chemokine genes in meta-analysis, but none of these variants survived the multiple testing correction (Additional file 2). The MAF of 71 common variants ranged from 0.01 to 0.5 in the combined control samples. Under the gene-only hypothesis and log additive model with an average population MAF of 0.1, the statistical power to detect an odds ratio (OR) value of 1.25 for a risk allele using the current sample size (1280 cases and 5044 controls) was above 88.8%. We observed a significant association of rs181868085 (*p* = 0.006, OR = 1.59) in *CXCL1* and rs2304973 (*p* = 0.045, OR = 0.84) in *CXCL16* with AD in the meta-analysis (Additional file 3). The OR values of these two SNPs indicated a consistent direction of genetic effect on disease risk between the Southern and the Eastern cohorts, although the association did not survive the multiple testing correction. Note that we observed a significant association between *CCL3* and AD in the Eastern cohort of Han Chinese, even after the Bonferroni correction (*p* = 4.35 × 10^−4^), but this association was weakened in the combined Han Chinese sample (*p* = 0.008; Additional file 1: Table S2), suggesting that population heterogeneity may exist even between our two cohorts under study. A reanalysis of the allele frequency data of 71 common variants in the newly published GWAS study [6] showed no association with AD in the European population (Additional file 1: Table S4).

Taken together, there seems to be no robust association of rare and common variants of chemokine genes with AD in both Han Chinese and European populations. The weak association between the chemokine gene and AD observed in the gene-based burden test might be caused by population substructure and stratification.

### MR analyses prioritized *CCL5* as a causal gene for AD

The MR analysis based on large-scale proteomic data of plasma chemokine levels [59, 60] and a genetic study of AD [3] showed that *CCL5* (*p* = 0.0055, β =  − 0.0667, Table 3) was causally linked to AD without the reverse causal effect (*p* = 0.7864, β = 0.0078). This result, together with the observation of altered expression of *CCL5* in AD patients and the mouse models, indicated that CCL5 dysregulations might be actively involved in the development of AD.

**Table 3:** Summary results of bi-direction MR estimates for causal effect between chemokines and Alzheimer’s disease

Exposure	Exposure ID	Outcome	Outcome ID	Nsnp	β	SE	*P*	OR	95% CI
CCL5	prot-a-409	Alzheimer's disease	ieu-b-2	29	− 0.0667	0.0240	**0.0055**	0.94	0.89–0.98
CXCL1	prot-b-16	Alzheimer's disease	ieu-b-2	8	− 0.0251	0.0353	0.4773	0.98	0.91–1.05
CXCL16	prot-a-745	Alzheimer's disease	ieu-b-2	27	− 0.0050	0.0246	0.8404	1.00	0.95–1.04
Alzheimer’s disease	ieu-b-2	CCL5	prot-a-409	50	0.0078	0.0290	0.7864	1.01	0.95–1.07
Alzheimer’s disease	ieu-b-2	CXCL1	Prot-b-16	32	0.0016	0.0380	0.9657	1.00	0.93–1.08
Alzheimer’s disease	ieu-b-2	CXCL16	prot-a-745	50	− 0.0404	0.0290	0.1636	0.96	0.91–1.02

*Nsnp* Number of SNPs used as instrumental variables with GWAS *p* < 1.0 × 10^−5^

*β* The effect size of exposure on outcome

*SE* Standard error of β

*P P* value of the causal effect inference by inverse-variance weighted (IVW) model

*OR* Odds ratio of the causal effect, *95% CI* 95% confidence interval of OR

## Discussion

Alteration of chemokines has been frequently reported to be involved in AD [28, 31, 32]. However, the exact mechanisms of the upregulation or downregulation of chemokine genes during the development of AD have not been sufficiently clarified, and this has led to a dispute regarding whether the chemokine expression alterations are the drivers, or by-products, of AD pathobiology. In this study, we aimed to determine the role of mRNA expression and genetic variations of 31 chemokine genes in AD, with an intention of defining the involvement of chemokines in AD. By integrating the genetic analyses, mRNA expression alterations, and pathological correlation in both AD patients and mouse models, we found an involvement of CXCL16 and CCL5 in the development of AD. This comprehensive analysis enabled us to provide a systematic view for understanding the roles of chemokines in the development of AD. First, the mRNA expression levels of a small proportion of chemokines under study were upregulated in brain regions both in AD patients (Table 1 and Fig. 2) and AD mice (Fig. 3). This result is consistent with previous reports for a higher level of proinflammatory chemokines in peripheral blood and brain tissues in AD patients [28, 64], and supports the significant role of chemokines in the neuroinflammatory process of AD. Second, we found that the upregulated chemokines were positively correlated with Aβ and tau pathology in AD mice (Fig. 3). This observation suggested an active role of chemokines in AD progression although their function in regulating Aβ or tau pathology remains to be determined [32]. Third, the MR analysis showed that *CCL5* was prioritized to be causally linked to AD, indicating that chemokine gene may be involved in AD in different ways.

We did not obtain a firm conclusion regarding the association of genetic variants of 31 chemokine genes with AD. Although we found five genes (*CCL3*, *CCR7*, *CCR9*, *CCRL2*, and *XCR1*) were nominally associated with AD in the combined Han Chinese sample in the gene-based burden test (Additional file 1: Table S2), none of the associations can be verified in the populations of European ancestry based on a data-mining of the ADSP dataset [44] (Additional file 1: Table S3). Instead, four other genes, *CCL2*, *CCR6*, *CX3CR1*, and *CXCL6*, were suggested to be associated with AD in the populations of European ancestry [44]. Our analysis of the potential association between rare or common variants and AD risk showed the same pattern of mixed signal for positive association. Therefore, we were unable to make a firm conclusion that genetic variants of the chemokine genes had a role in conferring genetic risk to AD, and the current weak association could be real or could be explained by different genetic structures between Asian and European populations. Independent validation analysis with a large sample size in populations of different ancestral origins is needed to clarify this issue.

The dysregulation of CCL5 and CXCL16 in AD patients at the transcriptional level deserved further attention. The CCL5 was reported to be upregulated in peripheral blood mononuclear cells of AD patients [29, 65], and Aβ42 treatment could increase the expression of CCL5 and its receptor CCR5 in peripheral mononuclear cells [66]. The upregulation of CCL5 in the AD brain may play a possible neuroprotective role [24], as soluble CCL5 activated by Aβ had an ameliorating effect on AD in mice by recruiting bone marrow-induced microglia immune response [67]. In this study, we found that CCL5 increases with AD development in both mouse models and patients, and an increased CCL5 level was causally associated with decreased AD risk in the MR analysis, collectively supporting the active role of CCL5 in AD and the potential utility of CCL5 as a therapeutic target.

Concerning the involvement of CXCL16 in AD, there were only a few reports available until very recently, Piehl et al. highlighted the CXCL16-CXCR6 axis in CSF of aged and AD brain [68]. These researchers found that CXCL16, derived from inflamed microglia and increased in CSF, activated the CD8^+^ T cell trafficking to the CSF through the CXCL16-CXCR6 pathway [68]. Note that dysregulation of CXCL16 was previously suggested as a possible mechanism of neurodegeneration in AD [69]. The consistent alteration pattern of CXCL16 in serum, CSF, and brain tissues of AD patients suggested that this chemokine might be used as a potential biomarker for monitoring AD development. A clinical observation study is needed to test this possibility.

This study has some limitations. First, the sample size used in genetic analyses was relatively small, and potential population stratification and different population structure might blur the potential association between chemokine genes and AD. Second, although we observed expression alterations of some chemokine genes in brain tissues of AD patients and the mouse models, we have not linked the expression changes to cell types and had no experimental data to discern its potential effect on cellular function of these affected cells. We also did not validate the causal role of the highlighted genes, such as *CCL5* and *CXCL16*, in animal models of AD [70]. Third, we only analyzed a proportion of chemokines in this study, and we could not exclude the possibility that other chemokine genes might have a prominent role in AD pathobiology. Nonetheless, the accumulating knowledge of chemokines’ roles in AD, as exemplified in this study with an intention of comprehensive integrative analysis, is undoubtedly essential for guiding the development of potential novel immunotherapies for AD.

## Conclusions

In short, through an extensive analysis of chemokine genes based on expressional, pathological, and genetic analysis data, we provide multiple lines of evidence to support the important role of chemokines CCL5 and CXCL16 in the development of AD. Further genetic studies with larger sample sizes and functional assays are needed to validate our conclusion and to depict the mechanisms of these two chemokines in AD pathogenesis.

## Supplementary Information

**Additional file 1: Fig. S1.** Up-regulation of mRNA expression of chemokine genes during aging in AD mouse models. **Fig. S2.** Correlation between the mRNA expression levels of chemokine genes with AD pathology in AD mouse models. **Table S1.** Sequence coverage of each gene in the targeted sequencing. **Table S2.** Results of SKAT-O analysis in Han Chinese with and without AD. **Table S3.** Results of gene-based burden test of chemokine genes from ADSP. **Table S4.** Association results of common variants in European population.

**Additional file 2.** Rare variants of 31 chemokine genes in Han Chinese.

**Additional file 3.** Association results of common variants in 31 chemokine genes in Han Chinese of AD.

## Data Availability

All data generated or analyzed during this study are included in this published article and its Additional files.

## Abbreviations

AD: Alzheimer’s disease
MCI: Mild cognitive impairment
MR: Mendelian randomization
GWAS: Genome-wide association study
WES: Whole-exome sequencing study
WGS: Whole-genome sequencing study
CNS: Central nervous system
CSF: Cerebrospinal fluid
SNP: Single nucleotide polymorphism
WILD: Wild-type mice
HO_TASTPM: Homozygous mutant human APP ^K670N/M671L^ and PSEN1 ^M146V^ mice
TAU: Mutant human MAPT ^P301L^ mice
ChinaMAP: China Metabolic Analytics Project
DSM-IV: Diagnostic and Statistical Manual of Mental Disorders IV
HWE: Hardy-Weinberg equilibrium
MAF: Minor allele frequency
LoF: Loss-of-function
M-CAP: Mendelian Clinically Applicable Pathogenicity
IVW: Inverse-variance weighted linear regression
ADSP: The Alzheimer’s Disease Sequencing Project
SKAT-O: The optimized sequence kernel association test
