# Supplementary material for: Convergent transcriptomic and genomic evidence supporting a dysregulation of CXCL16 and CCL5 in Alzheimer’s disease
Source: Alzheimers Res Ther. 2023 Jan 21;15:17. doi: 10.1186/s13195-022-01159-5 (PMC9863145; doi:10.1186/s13195-022-01159-5)
Supplement: Supplementary file 1 — Additional file 1: Fig. S1. Up-regulation of mRNA expression of chemokine genes during aging in AD mouse models. Fig. S2. Correlation between the mRNA expression levels of chemokine genes with AD pathology in AD mouse models. Table S1. Sequence coverage of each gene in the targeted sequencing. Table S2. Results of SKAT-O analysis in Han Chinese with and without AD. Table S3. Results of gene-based burden test of chemokine genes from ADSP. Table S4. Association results of common variants in European population. [file 13195_2022_1159_MOESM1_ESM.docx]

**Supplementary files**

This file contains two supplementary figures and four supplementary tables.


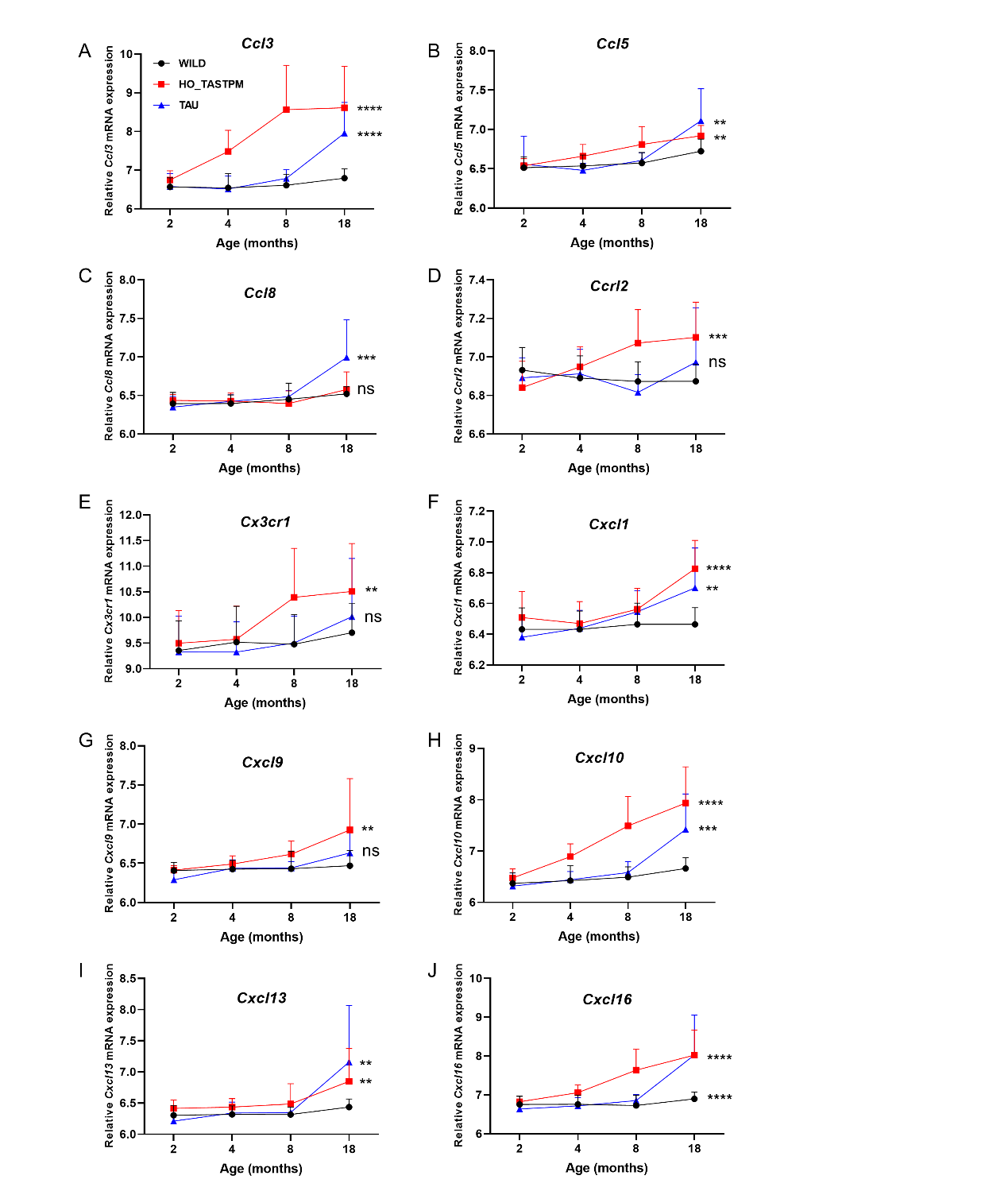


**Fig. S1** Up-regulation of mRNA expression of chemokine genes during aging in AD mouse models. **A-J** The age-related mRNA expression alteration of each of the 10 chemokine genes. Data were retrieved from Mouseac (www.mouseac.org) [1]. The age-related mRNA expression level was measured in 114 brain tissues from WILD mice, 44 brain tissues from HO_TASTPM mice, and 45 brain tissues from TAU mice at different life stages. Statistical differences were calculated by two-tailed student’s t-test. Error bars represent the population standard deviation. Ns, not significant; ***P* < 0.01; ****P* < 0.001; *****P* <0.0001.


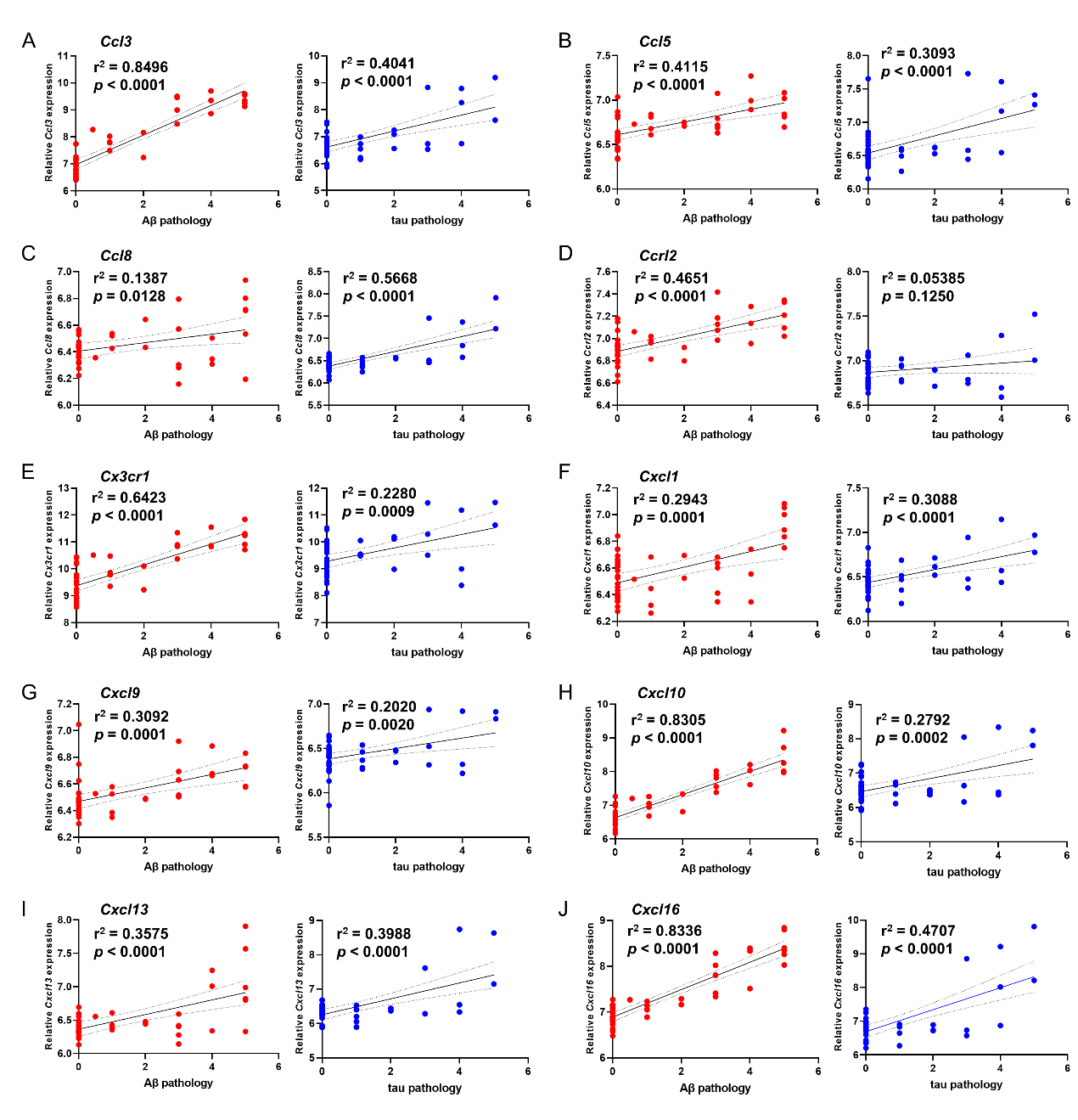


**Fig. S2** Correlation between the mRNA expression levels of chemokine genes with AD pathology in AD mouse models. Data were retrieved from Mouseac (www.mouseac.org) [1]. **A-J** shows the correlation between each of the chemokine gene and the Aβ and tau pathology. The scores of Aβ pathology were retrieved from 44 brain tissues of HO_TASTPM mice, and the scores of tau pathology were retrieved from 45 brain tissues of TAU mice. The correlations between mRNA expression levels and Aβ or tau pathology were measured using the Pearson correlation analysis. The solid and dashed lines represent the slope and the 95% confidence intervals in linear regression.

**Table S1** Sequence coverage of each gene in the targeted sequencing

| **Gene** | **Mean depth** | **10× Coverage (%)** | **20× Coverage (%)** | **30× Coverage (%)** | **50× Coverage (%)** |
| --- | --- | --- | --- | --- | --- |
| C-C motif chemokine ligand | | | | | |
| *CCL1* | 92.3 | 100 | 100 | 100 | 100 |
| *CCL2* | 94.1 | 100 | 100 | 100 | 97.0 |
| *CCL3* | 106.3 | 100 | 100 | 100 | 75.1 |
| *CCL5* | 118.7 | 100 | 100 | 100 | 100 |
| *CCL8* | 139.4 | 100 | 100 | 100 | 100 |
| *CCL11* | 134.2 | 100 | 100 | 100 | 100 |
| *CCL14* | 160.6 | 100 | 100 | 100 | 100 |
| *CCL15* | 146.2 | 100 | 100 | 100 | 98.4 |
| *CCL16* | 182.0 | 100 | 100 | 100 | 100 |
| C-C motif chemokine receptor | | | | | |
| *CCR1* | 229.2 | 100 | 100 | 100 | 100 |
| *CCR3* | 239.4 | 100 | 100 | 100 | 100 |
| *CCR5* | 250.2 | 100 | 100 | 100 | 100 |
| *CCR6* | 248.6 | 100 | 100 | 100 | 100 |
| *CCR7* | 139.7 | 100 | 100 | 100 | 100 |
| *CCR9* | 229.5 | 100 | 100 | 100 | 100 |
| *CCRL2* | 222.8 | 100 | 100 | 100 | 100 |
| C-X-C motif chemokine ligand | | | | | |
| *CXCL1* | 102.6 | 100 | 100 | 100 | 87.7 |
| *CXCL2* | 109.1 | 100 | 100 | 100 | 91.6 |
| *CXCL6* | 150.5 | 100 | 100 | 100 | 100 |
| *CXCL8* | 134.9 | 100 | 100 | 100 | 92.6 |
| *CXCL9* | 187.9 | 100 | 100 | 100 | 100 |
| *CXCL10* | 140.4 | 100 | 100 | 100 | 100 |
| *CXCL11* | 206.4 | 100 | 100 | 100 | 100 |
| *CXCL13* | 115.3 | 100 | 100 | 100 | 100 |
| *CXCL16* | 94.8 | 100 | 100 | 97.3 | 84.8 |
| C-X-C motif chemokine receptor | | | | | |
| *CXCR2* | 203.8 | 100 | 100 | 100 | 100 |
| *CXCR4* | 198.4 | 100 | 100 | 100 | 100 |
| *CXCR5* | 131.9 | 100 | 100 | 100 | 99.9 |
| *CXCR6* | 213.4 | 100 | 100 | 100 | 100 |
| C-X3-C motif chemokine receptor | | | | | |
| *CX3CR1* | 225.7 | 100 | 100 | 100 | 100 |
| X-C motif chemokine receptor | | | | | |
| *XCR1* | 171.3 | 100 | 100 | 100 | 100 |

The mean depth of a gene was calculated by an equation = total sequence data / gene length.

N× coverage was calculated by an equation = (the total number of nucleobases in a gene that was sequenced over N times / total gene length) × 100%.

**Table S2** Results of SKAT-O analysis in Han Chinese with and without AD

| **Gene** | **Southern cohort**  **(635 cases vs. 1507 controls)** | | |  | **Eastern cohort**  **(645 cases vs. 1507 controls)** | | |  | **Combined**  **(1280 cases vs. 1507 controls)** | | | |
| --- | --- | --- | --- | --- | --- | --- | --- | --- | --- | --- | --- | --- |
|  | **LoF** | **Possibly pathogenic** | **Missense** |  | **LoF** | **Possibly pathogenic** | **Missense** |  | **LoF** | **Possibly pathogenic** | **Missense** | |
| C-C motif chemokine ligand | | | | | | | | | | | |  |
| *CCL1* | NA | 0.648 (1) | 0.532 (2) |  | NA | 0.650 (1) | 0.515 (3) |  | NA | 0.730 (1) | 0.431 (3) | |
| *CCL2* | NA | NA | 0.780 (3) |  | NA | NA | 0.755 (2) |  | NA | NA | 0.798 (3) | |
| *CCL3* | NA | 1.000 (0) | 0.763 (4) |  | NA | 1.000 (0) | 4.351× 10^-4^ (5)* |  | NA | 1.000 (0) | 0.008 (6)* | |
| *CCL5* | NA | 0.648 (1) | 0.456 (5) |  | NA | 0.300 (2) | 0.591 (4) |  | NA | 0.752 (2) | 0.824 (6) | |
| *CCL8* | NA | NA | 0.148 (1) |  | NA | NA | 0.150 (1) |  | NA | NA | 0.105 (2) | |
| *CCL11* | NA | NA | 0.753 (2) |  | NA | NA | 0.755 (2) |  | NA | NA | 0.357 (2) | |
| *CCL14* | 1.000 (0) | NA | 0.532 (2) |  | 1.000 (0) | NA | 0.794 (2) |  | 1.000 (0) | NA | 0.608 (2) | |
| *CCL15* | NA | NA | 0.423 (3) |  | NA | NA | 0.222 (4) |  | NA | NA | 0.201 (5) | |
| *CCL16* | NA | NA | 0.125 (4) |  | NA | NA | 0.435 (5) |  | NA | NA | 0.110 (5) | |
| C-C motif chemokine receptor | | | | | | | | | | | |  |
| *CCR1* | NA | 0.532 (6) | 0.973 (9) |  | NA | 0.061 (9) | 0.362 (12) |  | NA | 0.187 (11) | 0.740 (14) | |
| *CCR3* | NA | 0.897 (6) | 0.930 (10) |  | NA | 0.463 (9) | 0.464 (14) |  | NA | 0.671 (11) | 0.853 (16) | |
| *CCR5* | NA | 0.798 (9) | 0.650 (17) |  | NA | 0.026 (12)* | 0.025 (19)* |  | NA | 0.121 (12) | 0.072 (20) | |
| *CCR6* | NA | 1.000 (0) | 0.772 (6) |  | NA | 0.150 (1) | 0.773 (6) |  | NA | 0.230 (1) | 0.847 (8) | |
| *CCR7* | 0.332 (1) | 0.004 (4)* | 0.142 (11) |  | 0.851 (1) | 1.000 (0) | 0.927 (6) |  | 0.418 (1) | 0.022 (4)* | 0.378 (12) | |
| *CCR9* | NA | 0.296 (2) | 0.009 (11)* |  | NA | 0.016 (5)* | 0.117 (13) |  | NA | 0.067 (6) | 0.015 (15)* | |
| *CCRL2* | NA | 0.592 (7) | 0.183 (15) |  | NA | 0.672 (7) | 0.089 (18) |  | NA | 0.876 (9) | 0.042 (20)* | |
| C-X-C motif chemokine ligand | | | | | | | | | | | |  |
| *CXCL1* | NA | 1.000 (0) | 0.456 (4) |  | NA | 1.000 (0) | 0.755 (2) |  | NA | 1.000 (0) | 0.524 (4) | |
| *CXCL2* | 0.385 (1) | NA | 0.290 (3) |  | 0.387 (1) | NA | 0.288 (6) |  | 0.176 (1) | NA | 0.457 (6) | |
| *CXCL6* | NA | NA | 0.057 (2) |  | NA | NA | 0.109 (3) |  | NA | NA | 0.118 (3) | |
| *CXCL8* | NA | NA | 0.456 (5) |  | NA | NA | 0.562 (4) |  | NA | NA | 0.457 (6) | |
| *CXCL9* | NA | NA | 0.516 (3) |  | NA | NA | 0.526 (3) |  | NA | NA | 0.413 (3) | |
| *CXCL10* | NA | 0.648 (1) | 0.853 (2) |  | NA | 0.650 (1) | 0.853 (2) |  | NA | 0.730 (1) | 0.722 (2) | |
| *CXCL11* | 1.000 (0) | 0.532 (2) | 0.301 (7) |  | 1.000 (0) | 0.526 (3) | 0.761 (8) |  | 1.000 (0) | 0.686 (3) | 0.320 (8) | |
| *CXCL13* | NA | NA | 0.772 (2) |  | NA | NA | 0.308 (3) |  | NA | NA | 0.736 (3) | |
| *CXCL16* | NA | 0.408 (1) | 0.232 (9) |  | NA | 0.904 (1) | 0.277 (9) |  | NA | 0.502 (1) | 0.178 (11) | |
| C-X-C motif chemokine receptor | | | | | | | | | | | |  |
| *CXCR2* | NA | 0.780 (3) | 0.378 (12) |  | NA | 0.755 (2) | 0.710 (10) |  | NA | 0.798 (3) | 0.837 (13) | |
| *CXCR4* | NA | 1.000 (0) | 0.739 (5) |  | NA | 0.150 (1) | 0.120 (5) |  | NA | 0.230 (1) | 0.329 (7) | |
| *CXCR5* | NA | 0.296 (2) | 0.872 (6) |  | NA | 0.121 (3) | 0.559 (8) |  | NA | 0.234 (4) | 0.934 (9) | |
| *CXCR6* | NA | 0.780 (3) | 0.666 (4) |  | NA | 0.755 (2) | 0.245 (5) |  | NA | 0.798 (3) | 0.603 (6) | |
| C-X3-C motif chemokine receptor | | | | | | | | | | | |  |
| *CX3CR1* | NA | 0.662 (3) | 0.758 (13) |  | NA | 0.469 (2) | 0.362 (10) |  | NA | 0.496 (3) | 0.629 (14) | |
| X-C motif chemokine receptor | | | | | | | | | | | |  |
| *XCR1* | NA | 0.275 (8) | 0.277 (18) |  | NA | 0.054 (8) | 0.106 (17) |  | NA | 0.031 (9)* | 0.069 (20) | |

Numbers of tested variants are shown in parentheses and association with nominal significance (*p* < 0.05) was marked with a “*”.

LoF, loss-of-function variants.

**Table S3** Results of gene-based burden test of chemokine genes from ADSP [2]

| **Gene** | **nSNPs** | **cMAC_all** | **p_m0** | **p_m1** | **p_m2** |
| --- | --- | --- | --- | --- | --- |
| C-C motif chemokine ligand | | | | | |
| *CCL1* | 10 | 15 | 0.508 | 0.580 | 0.516 |
| *CCL11* | 14 | 42 | 0.265 | 0.292 | 0.354 |
| *CCL14* | 21 | 1766 | 0.073 | 0.114 | 0.160 |
| *CCL15* | 11 | 1058 | 0.489 | 0.462 | 0.731 |
| *CCL16* | 17 | 382 | 0.651 | 0.401 | 0.313 |
| *CCL2* | 5 | 24 | 0.064 | **0.033** | **0.030** |
| *CCL3* | 14 | 81 | 0.144 | 0.374 | 0.294 |
| *CCL5* | 10 | 21 | 0.792 | 0.469 | 0.541 |
| *CCL8* | 14 | 559 | 0.175 | 0.348 | 0.413 |
| C-C motif chemokine receptor | | | | | |
| *CCR1* | 26 | 111 | 0.473 | 0.451 | 0.602 |
| *CCR3* | 36 | 535 | 0.662 | 0.787 | 0.811 |
| *CCR5* | 57 | 744 | 0.452 | 0.117 | 0.160 |
| *CCR6* | 24 | 31 | **0.015** | 0.058 | 0.147 |
| *CCR7* | 25 | 721 | 0.985 | 0.704 | 0.755 |
| *CCR9* | 32 | 360 | 0.239 | 0.601 | 0.333 |
| *CCRL2* | 46 | 798 | 0.472 | 0.941 | 0.912 |
| C-X-C motif chemokine ligand | | | | | |
| *CXCL1* | 12 | 60 | 0.331 | 0.064 | 0.053 |
| *CXCL10* | 10 | 136 | 0.699 | 0.095 | 0.107 |
| *CXCL11* | 16 | 179 | 0.104 | 0.645 | 0.445 |
| *CXCL13* | 11 | 16 | 0.300 | 0.407 | 0.614 |
| *CXCL16* | 21 | 273 | 0.322 | 0.491 | 0.487 |
| *CXCL2* | 14 | 490 | 0.681 | 0.350 | 0.286 |
| *CXCL6* | 13 | 107 | **0.020** | 0.101 | 0.115 |
| *CXCL9* | 11 | 33 | 0.442 | 0.804 | 0.864 |
| C-X-C motif chemokine receptor | | | | | |
| *CXCR2* | 29 | 154 | 0.282 | 0.254 | 0.349 |
| *CXCR4* | 23 | 40 | 0.665 | 0.948 | 0.830 |
| *CXCR5* | 26 | 44 | 0.752 | 0.432 | 0.348 |
| *CXCR6* | 15 | 163 | 0.990 | 0.599 | 0.601 |
| C-X3-C motif chemokine receptor | | | | | |
| *CX3CR1* | 27 | 436 | **0.011** | **0.013** | **0.015** |
| X-C motif chemokine receptor | | | | | |
| *XCR1* | 28 | 188 | 0.542 | 0.811 | 0.771 |

Data for *CXCL8* was not available in this study. nSNPs, number of SNPs tested; cMAC_all, cumulative minor allele count in all samples including 5740 AD cases and 5096 cognitively normal controls in discovery stage; p_mo, *p* values were adjusted for PCs and sequencing center; p_m1, *p* values were adjusted for sex and age at diagnosis or last follow-up in addition to those included in mo; p_m2, *p* values were adjusted for *APOE* ε4 & ε2 dosages in addition to those included in m1.

**Table S4** Association results of common variants in European population

| **Gene** | **Variant_id** | ***P*** | **Chr** | **Location** | **Allele** | **Effect_allele_freq** | **OR** | **ci_lower** | **ci_upper** | **beta** | **se** |
| --- | --- | --- | --- | --- | --- | --- | --- | --- | --- | --- | --- |
| *CCL2* | rs28730833 | 0.2523 | 17 | 34255475 | A/T | 0.0129 | 1.045 | 0.969 | 1.126 | 0.0436 | 0.0381 |
| *CCL2* | rs4586 | 0.1808 | 17 | 34256250 | T/C | 0.6398 | 1.011 | 0.995 | 1.028 | 0.0113 | 0.0084 |
| *CCL2* | rs13900 | 0.5038 | 17 | 34256892 | T/C | 0.2693 | 0.994 | 0.976 | 1.012 | -0.0061 | 0.0091 |
| *CCL3* | rs1130371 | 0.7399 | 17 | 36089191 | A/G | 0.2311 | 0.997 | 0.978 | 1.016 | -0.0032 | 0.0098 |
| *CCL8* | rs41410552 | 0.7661 | 17 | 34319112 | A/G | 0.107 | 1.004 | 0.978 | 1.03 | 0.0039 | 0.0132 |
| *CCL8* | rs1133763 | 0.6492 | 17 | 34320812 | A/C | 0.8455 | 0.995 | 0.973 | 1.017 | -0.0051 | 0.0113 |
| *CCL11* | rs1129844 | 0.2609 | 17 | 34285875 | A/G | 0.1834 | 1.012 | 0.991 | 1.033 | 0.0118 | 0.0105 |
| *CCL14* | rs75238886 | 0.9026 | 17 | 35986576 | A/G | 0.0317 | 1.003 | 0.958 | 1.049 | 0.0028 | 0.0231 |
| *CCL14* | rs113937434 | 0.9316 | 17 | 35986638 | A/G | 0.0313 | 1.002 | 0.957 | 1.049 | 0.002 | 0.0232 |
| *CCL15* | rs854625 | 0.7695 | 17 | 36001422 | A/G | 0.0451 | 0.994 | 0.955 | 1.034 | -0.006 | 0.0203 |
| *CCL16* | rs79254649 | 0.3018 | 17 | 35977569 | A/C | 0.0242 | 0.971 | 0.918 | 1.027 | -0.0295 | 0.0286 |
| *CCL16* | rs11080369 | 0.7433 | 17 | 35978128 | A/C | 0.9292 | 0.995 | 0.964 | 1.026 | -0.0052 | 0.0159 |
| *CCL16* | rs114853983 | 0.2714 | 17 | 35978326 | T/C | 0.0241 | 0.969 | 0.916 | 1.025 | -0.0315 | 0.0287 |
| *CCR1* | rs34423195 | 0.676 | 3 | 46208231 | A/G | 0.9218 | 1.006 | 0.977 | 1.037 | 0.0064 | 0.0153 |
| *CCR1* | rs3181080 | 0.6745 | 3 | 46208438 | A/T | 0.9218 | 1.006 | 0.977 | 1.037 | 0.0064 | 0.0153 |
| *CCR3* | rs9853223 | 0.5162 | 3 | 46242602 | A/G | 0.4376 | 0.995 | 0.979 | 1.011 | -0.0053 | 0.0082 |
| *CCR3* | rs4987053 | 0.3866 | 3 | 46265209 | T/C | 0.929 | 1.014 | 0.983 | 1.046 | 0.0137 | 0.0159 |
| *CCR5* | rs1800452 | 0.9312 | 3 | 46373570 | A/G | 0.001 | 0.985 | 0.706 | 1.376 | -0.0147 | 0.1704 |
| *CCR6* | rs1012656 | 0.865 | 6 | 167111815 | C/G | 0.4645 | 1.001 | 0.986 | 1.017 | 0.0014 | 0.0081 |
| *CCR6* | rs3093009 | 0.3378 | 6 | 167135989 | A/G | 0.7813 | 1.01 | 0.99 | 1.03 | 0.0096 | 0.01 |
| *CCR6* | rs3093007 | 0.4998 | 6 | 167136287 | T/C | 0.8149 | 1.007 | 0.987 | 1.028 | 0.0072 | 0.0106 |
| *CCR6* | rs2071171 | 0.489 | 6 | 167136554 | T/C | 0.6183 | 0.994 | 0.978 | 1.011 | -0.0058 | 0.0084 |
| *CCR7* | rs588019 | 0.7504 | 17 | 40558855 | A/G | 0.0732 | 0.995 | 0.963 | 1.028 | -0.0053 | 0.0167 |
| *CCRL2* | rs11266744 | 0.19 | 3 | 46408487 | A/C | 0.6008 | 0.989 | 0.973 | 1.005 | -0.0109 | 0.0083 |
| *CCRL2* | rs3204849 | 0.1942 | 3 | 46408579 | A/T | 0.3961 | 1.011 | 0.995 | 1.027 | 0.0108 | 0.0083 |
| *CCRL2* | rs3204850 | 0.8376 | 3 | 46408806 | A/G | 0.9152 | 1.003 | 0.975 | 1.032 | 0.003 | 0.0147 |
| *CXCL1* | rs11547681 | 0.1551 | 4 | 73869433 | T/G | 0.1814 | 0.985 | 0.965 | 1.006 | -0.0151 | 0.0106 |
| *CXCL1* | rs2071425 | 0.1036 | 4 | 73869527 | A/G | 0.7924 | 1.017 | 0.997 | 1.037 | 0.0165 | 0.0101 |
| *CXCL1* | rs7656335 | 0.5066 | 4 | 73869807 | C/G | 0.0517 | 0.988 | 0.952 | 1.025 | -0.0124 | 0.0187 |
| *CXCL1* | rs4074 | 0.05719 | 4 | 73870427 | A/G | 0.3681 | 0.984 | 0.967 | 1.001 | -0.0163 | 0.0086 |
| *CXCL9* | rs2276886 | 0.7759 | 4 | 76007275 | T/C | 0.2484 | 0.997 | 0.979 | 1.016 | -0.0027 | 0.0094 |
| *CXCL9* | rs2276885 | 0.667 | 4 | 76007576 | A/G | 0.8049 | 0.996 | 0.976 | 1.016 | -0.0044 | 0.0102 |
| *CXCL10* | rs3921 | 0.7409 | 4 | 76021790 | C/G | 0.464 | 0.997 | 0.982 | 1.013 | -0.0027 | 0.0081 |
| *CXCL11* | rs6532111 | 0.764 | 4 | 76034761 | T/C | 0.4637 | 0.998 | 0.982 | 1.014 | -0.0024 | 0.0081 |
| *CXCL11* | rs6819597 | 0.7659 | 4 | 76036018 | T/C | 0.5362 | 1.002 | 0.987 | 1.018 | 0.0024 | 0.0081 |
| *CXCL13* | rs17002743 | 0.3168 | 4 | 77610778 | T/C | 0.004 | 0.906 | 0.747 | 1.099 | -0.0986 | 0.0985 |
| *CXCL16* | rs1051009 | 0.9637 | 17 | 4734591 | A/G | 0.3402 | 1 | 0.983 | 1.017 | -4.00× 10^-4^ | 0.0087 |
| *CXCL16* | rs3744700 | 0.2228 | 17 | 4734715 | T/G | 0.3496 | 1.011 | 0.994 | 1.028 | 0.0106 | 0.0087 |
| *CXCL16* | rs1876444 | 0.7667 | 17 | 4735189 | T/C | 0.4479 | 0.998 | 0.982 | 1.014 | -0.0024 | 0.0082 |
| *CXCL16* | rs2277680 | 0.5392 | 17 | 4735268 | A/G | 0.4295 | 0.995 | 0.979 | 1.011 | -0.005 | 0.0082 |
| *CXCL16* | rs1050998 | 0.5711 | 17 | 4735442 | A/G | 0.5679 | 1.005 | 0.989 | 1.021 | 0.0046 | 0.0082 |
| *CXCL16* | rs1050997 | 0.3679 | 17 | 4738460 | T/C | 0.7611 | 1.009 | 0.99 | 1.029 | 0.0089 | 0.0099 |
| *CXCL16* | rs2250333 | 0.1863 | 17 | 4738774 | A/G | 0.1824 | 0.986 | 0.964 | 1.007 | -0.0146 | 0.011 |
| *CXCL16* | rs2304973 | 0.7027 | 17 | 4738927 | A/G | 0.0822 | 0.994 | 0.965 | 1.025 | -0.0059 | 0.0154 |
| *CXCR2* | rs4674259 | 0.05653 | 2 | 218126282 | A/G | 0.5302 | 0.985 | 0.969 | 1 | -0.0155 | 0.0081 |
| *CXCR2* | rs11574750 | 0.115 | 2 | 218135569 | T/C | 0.046 | 0.97 | 0.933 | 1.008 | -0.0307 | 0.0195 |
| *CXCR2* | rs2230054 | 0.2085 | 2 | 218135587 | T/C | 0.4828 | 0.99 | 0.974 | 1.006 | -0.0102 | 0.0081 |
| *CXCR4* | rs2228014 | 0.8075 | 2 | 136115514 | A/G | 0.0397 | 1.005 | 0.964 | 1.048 | 0.0052 | 0.0212 |
| *CXCR5* | rs2276344 | 0.5039 | 11 | 118883809 | A/T | 0.006 | 1.039 | 0.929 | 1.162 | 0.0381 | 0.057 |
| *CXCR5* | rs10892307 | 0.266 | 11 | 118883940 | C/G | 0.8405 | 0.988 | 0.966 | 1.01 | -0.0124 | 0.0112 |
| *CXCR5* | rs598207 | 0.1642 | 11 | 118894558 | C/G | 0.2674 | 1.013 | 0.995 | 1.031 | 0.0129 | 0.0092 |
| *CXCR6* | rs2234358 | 0.9427 | 3 | 45947552 | T/G | 0.4966 | 0.999 | 0.984 | 1.015 | -6.00 × 10^-4^ | 0.0081 |
| *CX3CR1* | rs3732378 | 0.7644 | 3 | 39265671 | A/G | 0.1629 | 0.997 | 0.976 | 1.018 | -0.0033 | 0.0109 |
| *CX3CR1* | rs3732379 | 0.5591 | 3 | 39265765 | T/C | 0.2792 | 1.005 | 0.988 | 1.023 | 0.0052 | 0.009 |
| *CX3CR1* | rs11715522 | 0.9979 | 3 | 39281672 | A/C | 0.6025 | 1 | 0.984 | 1.017 | 0 | 0.0084 |
| *XCR1* | rs2230322 | 0.2389 | 3 | 46021837 | T/C | 0.893 | 1.016 | 0.99 | 1.043 | 0.0157 | 0.0133 |
| *XCR1* | rs71327010 | 0.255 | 3 | 46027343 | T/G | 0.1035 | 0.985 | 0.959 | 1.011 | -0.0153 | 0.0135 |
| *XCR1* | rs7623476 | 0.1307 | 3 | 46027351 | A/G | 0.7796 | 0.985 | 0.967 | 1.004 | -0.0148 | 0.0098 |

Summary statistics are available through the National Human Genome Research Institute-European Bioinformatics Institute GWAS catalog under accession number GCST90027158 (https://www.ebi.ac.uk/gwas/) [3].

Thirteen common variants (rs193057414, rs74842203, rs117290001, rs181868085, rs3732380, rs137916685, rs70937035, rs151132172, rs17038679, rs61758325, rs41339751, rs75158174, rs55901334) have no data in this GWAS study.

Chr, chromosome; Location, base pair location in GRCh38; Allele, effect allele/other allele; OR, odds ratio of the effect allele; ci, confidence interval of OR; se, standard error.

**References**

1. Matarin M, Salih DA, Yasvoina M, Cummings DM, Guelfi S, Liu W, et al. A genome-wide gene-expression analysis and database in transgenic mice during development of amyloid or tau pathology*.* Cell Rep. 2015;10:633-44.

2. Bis JC, Jian X, Kunkle BW, Chen Y, Hamilton-Nelson KL, Bush WS, et al. Whole exome sequencing study identifies novel rare and common Alzheimer's-Associated variants involved in immune response and transcriptional regulation*.* Mol Psychiatry. 2020;25:1859-75.

3. Bellenguez C, Küçükali F, Jansen IE, Kleineidam L, Moreno-Grau S, Amin N, et al. New insights into the genetic etiology of Alzheimer's disease and related dementias*.* Nat Genet. 2022;54:412-36.
